# Patients with an Achilles tendon re-rupture have long-term functional deficits in function and worse patient-reported outcome than primary ruptures

**DOI:** 10.1007/s00167-018-4952-0

**Published:** 2018-04-24

**Authors:** Olof Westin, Katarina Nilsson Helander, Karin Grävare Silbernagel, Kristian Samuelsson, Annelie Brorsson, Jón Karlsson

**Affiliations:** 1 Department of Orthopaedics, Institute of Clinical Sciences, The Sahlgrenska Academy, University of Gothenburg, Gothenburg, Sweden; 2 Department of Orthopaedics, Sahlgrenska University Hospital, Mölndal, Sweden; 3 Hallands Sjukhus, Kungsbacka, Sweden; 4 IFK Kliniken Rehab, Gothenburg, Sweden; 5 Department of Physical Therapy, University of Delaware, Newark, DE USA

**Keywords:** Achilles tendon, Re-ruptures, Outcome, Tendon length, Long-term follow-up, Primary ruptures, Functional results

## Abstract

**Purpose:**

The aim of this study was to perform a long-term follow-up of patients treated for an Achilles tendon re-rupture, using established outcome measurements for tendon structure, lower extremity function and symptoms, and to compare the results with those for the uninjured side. A secondary aim was to compare the outcome with that of patients treated for primary ruptures. The hypotheses were that patients with a re-rupture recover well, and have similar long-term outcome as primary ruptures.

**Methods:**

Twenty patients (4 females) with a mean (SD) age of 44 (10.9) years, ranging from 24 to 64, were included. The patients were identified by reviewing the medical records of all Achilles tendon ruptures at Sahlgrenska University Hospital and Kungsbacka Hospital, Sweden, between 2006 and 2016. All patients received standardised surgical treatment and rehabilitation. The mean (SD) follow-up was 51 (38.1) months. A test battery of validated clinical and functional tests, patient-reported outcome measurements and measurements of tendon elongation were performed at the final follow-up. This cohort was then compared with the 2-year follow-up results from a previous randomised controlled trial of patients treated for primary Achilles tendon rupture.

**Results:**

There were deficits on the injured side compared with the healthy side in terms of heel-rise height (11.9 versus 12.5 cm, *p* = 0.008), repetitions (28.5 versus 31.7, *p* = 0.004) and drop-jump height (13.2 versus 15.1 cm, *p* = 0.04).  There was a significant difference in calf circumference (37.1 versus 38.4 cm, *p* =< 0.001) and ankle dorsiflexion on the injured side compared with the healthy side (35.3° versus 40.8°, *p* = 0.003). However, no significant differences were found in terms of tendon length 22.5 (2.5) cm on the injured side and 21.8 (2.8) cm on the healthy side. Compared with primary ruptures, the re-rupture cohort obtained significantly worse results for the Achilles tendon total rupture score, with a mean of 78 (21.2) versus 89.5 (14.6) points, (*p* = 0.007). The re-ruptures showed a higher mean LSI heel-rise height, 94.7% (9.3%) versus 83.5% (11.7%) (*p* = < 0.0001), and superior mean LSI eccentric-concentic power, 110.4% (49.8%) versus 79.3% (21%) (*p* = 0.001), than the primary ruptures.

**Conclusion:**

The results of this study indicate that patients with an Achilles tendon re-rupture had continued symptoms and functional deficits on the injured side, after a long-term follow-up. Patients with an Achilles tendon re-rupture had worse patient-reported outcomes but similar or superior functional results compared with patients with primary ruptures.

**Level of evidence:**

Case series, Level IV.

## Introduction

Acute Achilles tendon rupture is a common injury, with increasing incidence [5], and often leads to significant morbidity [5]. Several randomised controlled trials (RCT) have been conducted with the aim of identifying the optimal management strategy, in terms of both initial treatment (surgical versus non-surgical intervention) and rehabilitation [9, 14, 20, 30]. The primary outcome for the RCTs that have been conducted has historically been the re-rupture rate, indicating treatment failure. A meta-analysis conducted by Deng et al. [4] reported that the re-rupture rate in surgically treated patients was 3.7% compared with 9.8% in non-surgically managed patients. However, with surgical management, there are risks of sural nerve damage and wound infections [21]. A deep infection can lead to a prolonged hospital stay and, in severe cases, even requires advanced reconstructive surgery and may ultimately be devastating for the individual patient [21, 31, 32]. Most patients that sustain a re-rupture need to undergo surgical reconstruction and often have a prolonged recovery, with a negative effect on both work and leisure activities [15]. However, little is known about the effect of a re-rupture on patient outcome in the long-term.

There are few previous studies that have evaluated the long-term outcome of an Achilles tendon re-rupture and all these studies have included fewer than 20 patients [11, 21, 22, 25]. A total of 44 patients, from a heterogeneous study population, have been evaluated in all four studies, using non-validated outcome measurements, making it difficult to draw any strong conclusions from these data. All four studies found that patients have reduced calf muscle strength on the injured side compared with the contralateral healthy side at follow-up. These studies only used the uninjured side as a control and no previous study has made a comparison with the long-term follow-up results of primary ruptures.

The aim of this study was to perform a long-term follow-up of patients with an Achilles tendon re-rupture using established outcome measurements for tendon structure, lower extremity function and symptoms, and to compare the results with those for the uninjured side. Using patients treated both surgically and non-surgically for their primary rupture. A secondary aim of the study was to compare the re-rupture outcome with that of patients treated for primary ruptures. The hypotheses were that patients with a re-rupture recover well and have similar long-term outcome as primary ruptures.

## Materials and methods

**Fig. 1:**
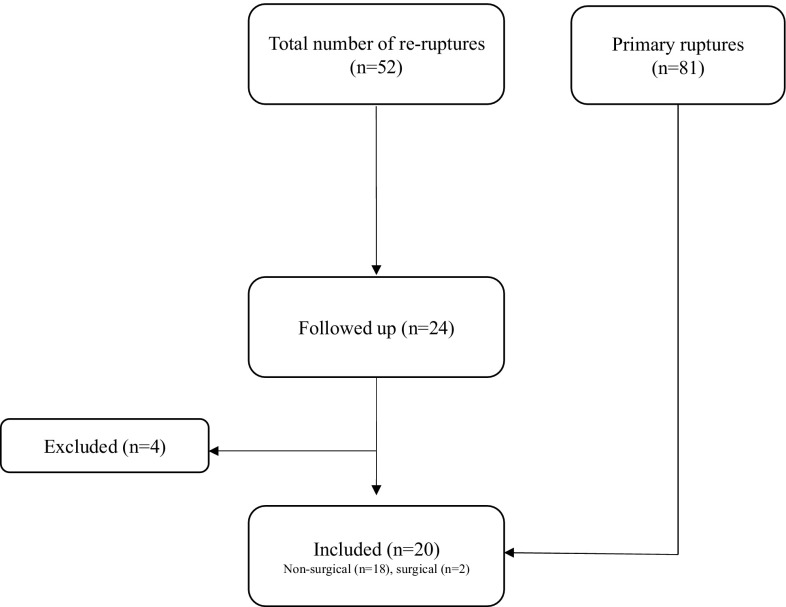
Flow diagram of the study

The patients were identified by reviewing the medical records of all Achilles tendon ruptures at Sahlgrenska University Hospital and Kungsbacka Hospital, Sweden, between 2006 and 2016. The inclusion criterion was any patient with a unilateral Achilles tendon re-rupture within the last 10 years. The exclusion criteria were age more than 70, diabetes, other injuries affecting the limb, neuromuscular disease, peripheral vascular disease, immunosuppressive therapy and inability to perform the follow-up evaluation. The re-ruptures were then compared with 2-year follow-up data from a previous randomised controlled trial, where surgical and non-surgical treatments were evaluated [14] (Fig. 1).

### Patients

Fifty-two patients were identified and 24 of them agreed to participate. Four patients were excluded because they did not fit the study inclusion criterion. One had bilateral ruptures, two had chronic ruptures and one had a recent ankle fracture, which made the evaluation impossible. A total of 20 patients (16 males) with a mean (SD) age of 44 (10.9) years were included in the study (Table 1). All the patients gave their written informed consent prior to participation in the study.

**Table 1:** Patient baseline characteristics

(A) Re-ruptures	Total (*n* = 20)	Male (*n* = 16)	Female (*n* = 4)
Age (years)	44.0 (10.9)	44.6 (9.8)	41.5 (16.2)
	(38.9; 49.0)	(39.4; 49.8)	(15.7; 67.3)
	*n* = 20	*n* = 16	*n* = 4
Weight (kg)	80.4 (10.8)	83.5 (9.6)	68.0 (4.2)
	(75.4; 85.5)	(78.4; 88.6)	(61.2; 74.7)
	*n* = 20	*n* = 16	*n* = 4
Height (cm)	179.1 (7.3)	181.8 (5.2)	168.3 (3.3)
	(175.7; 182.5)	(179.0; 184.6)	(163.0; 173.5)
	*n* = 20	*n* = 16	*n* = 4
BMI (kg/m^2^)	25.1 (3.1)	25.3 (3.3)	24.0 (2.1)
	(23.6; 26.5)	(23.6; 27.1)	(20.7; 27.4)
	*n* = 20	*n* = 16	*n* = 4
Smoking	2 (10.0%)	2 (12.5%)	0 (0.0%)

(B) Primary ruptures	Total (*n* = 81)	Male (*n* = 67)	Female (*n* = 14)
Age (years)	41.8 (9)	41.6 (9.4)	43.3 (7.5)
	(39.9; 43.8)	(39.4; 43.8)	(39; 47.6)
	*n* =81	*n* = 67	*n* = 14
Weight (kg)	84.8 (13.1)	88 (10.7)	68.1 (12.2)
	(81.9; 87.6)	(85.5; 90.5)	(61.1; 75.2)
	*n* = 81	*n* = 67	*n* = 14
Height (cm)	178.2 (9)	181.1 (6.3)	163.4 (3.3)
	(176.3; 180.2)	(179.7; 182.6)	(160; 166.8)
	*n* = 81	*n* = 67	*n* = 14
BMI (kg/m^2^)	26.6 (3.1)	26.8 (2.9)	25.5 (4.5)
	(26; 27.3)	(26.1; 27.5)	(22.9; 28.1)
	*n* = 81	*n* = 67	*n* = 14

For categorical variables *n* (%) is presented. For continuous variables mean (SD)/(95% CI for mean)/*n* = is presented

Ethical approval was granted by the regional ethical review board in Sweden, (DNR 058-14).

The initial treatment of the re-rupture group was mixed, with non-surgical (*n* = 18), with a cast for 2 weeks, followed by early weight-bearing in a walker brace for 6 weeks, and surgical (*n* = 2), with a below-the-knee cast for 2 weeks, followed by a walker brace for another 6 weeks. The mean follow-up from the time of the index injury for re-ruptures were 50.9 (38.1) months.

### Demographics

#### Treatment of re-rupture

The surgical technique and rehabilitation have previously been published [15]. To summarise, with the patient in a prone position, an approximately 20 cm long central incision, slightly medially curved distally to avoid damaging the sural nerve, was made. After debridement, an end-to-end suture using a modified Kessler suturing technique was performed [8, 15]. A free gastrocnemius aponeurosis flap was then prepared, the length and width depending on the tendon gap. The free flap was sutured over the gap and the defect in the aponeurosis was closed. Postoperatively, a below-the-knee cast was used with the foot in the equinus position. After 3–6 weeks, the cast was replaced by a movable walker brace (Don-Joy ROM-Walker), with gradually reduced plantar flexion. The total cast/brace period was 8 weeks. Full weight-bearing was started at 6–10 weeks, depending on the possibility of reaching the neutral position. The rehabilitation was supervised by an experienced physiotherapist.

### Follow-up evaluation

One experienced physiotherapist conducted all the follow-up examinations. The evaluation consisted of patient-reported outcome measurements (PROMs), a physical examination including clinical measurements, ultrasound imaging to measure tendon length [28] and a validated functional test battery to evaluate lower leg function [27].

### Patient-reported outcome

#### Achilles tendon total rupture score

The Achilles tendon total rupture score (ATRS) is a reliable, validated patient-reported outcome measurement [16]. Ten questions are included, each scored from 0 to 10, resulting in a total sum of 100, which means the patient has recovered completely, while a lower score indicates more symptoms and greater limitations in function [16].

#### Physical activity scale

The physical activity scale (PAS) is a score from 1 to 6, where 1 equals no physical activity, while a score of 6 means heavy physical exercise several times a week [6, 24].

#### Foot and ankle outcome score

All five subscales of the foot and ankle outcome score (FAOS) [23] were used in this study. The FAOS is a reliable, validated score that measures activities of daily living (ADL), function in sports and recreation, foot- and ankle-related quality of life (QOL), foot and ankle pain and other symptoms. The subscales range from 0 to 100, with 100 indicating full recovery and no foot- and ankle-related problems, whereas a score of 0 indicates severe problems.

#### Recovery percentage

Patients were asked to estimate their recovery (in percent) compared with their pre-injury function (100% representing fully recovered to pre-injury level) [10].

### Functional test

The MuscleLab^®^ (Ergotest Technology, Oslo, Norway) measurement system was used for the functional evaluation [27]. MuscleLab^®^ is a data collection unit to which various sensors can be connected. The functional tests have been used in several studies to evaluate outcome in patients after Achilles tendon injury [14, 20, 27].

The heel-rise endurance test was conducted with the patient standing on a box with a 10° incline and a spring-loaded string of the linear encoder was attached to the heel of the participant’s shoe. The patient was then instructed to perform as many single-leg standing heel-rises as possible at a pace of 30 reps/min guided by a metronome. The total amount of work was documented (body weight × total distance) in joules, as well as the number of repetitions and the height of each heel rise. The maximum height achieved was also documented.

In all the jump tests, patients were able to familiarise themselves with the jumping technique before testing. The first of the jump tests was a counter-movement jump (CMJ). Here, the patients were asked quickly to bend their knee and then make a vertical jump as high as possible. Three jumps were made on each side and the highest value was recorded and used in the analysis. Second, a drop CMJ was performed. The participants were asked to stand on a 20-cm high box on one leg, then jump down onto the floor and, as soon as they hit the ground, jump vertically as high as possible. The maximum jumping height (in cm) was used for data analysis. Hopping was the final jump test; this was done by getting the participants to perform a rhythmical jump similar to skipping with a rope, one leg at a time. Twenty jumps were used to calculate the mean hopping height and the plyometric quotient (flight time/contact time) was used for data analysis.

The calf muscle strength test was performed with the patient standing in a weight-training machine and performing a single-leg heel rise. The patients were told to perform the heel rise as quickly and forcefully as possible and the knee was not allowed to flex more than 20°. It was then repeated three times with the patient’s body weight plus 13 kg for the first test. The weight was progressively increased in 10-kg increments until a decrease in the patients’ power output was noted. The max power in watts was recorded and used for analysis.

### Clinical measurements

#### Achilles tendon resting angle

The Achilles tendon resting angle (ATRA) was described and validated by Carmont et al. [3]. It was performed with the participant in the prone position, with the knee flexed at 90°. The patients were instructed to relax their leg. A goniometer was placed with one arm along the shaft of the fibula, aligned with the centre of the fibula head. The other arm was aligned with the head of the fifth metatarsal. The angle between the arms was documented and used for analysis.

#### Dorsiflexion range of motion and calf circumference

The patients’ ability to dorsiflex their ankle joint was measured in standing, with both knee straight and bent, using an inclinometer, with the technique described by Munteanu et al. [13].

The calf circumference was measured at the largest area of the calf muscle with a standard tape measured in 1 mm increments [3]. The patient was in a prone position with the knee flexed at 90°. Repeated measurements were made until the same value was found for successive measurements, according to the method by Carmont et al. [3].

#### Tendon length measurement

The LOGIQ e US (GE Healthcare) system with a wide-band linear array probe (5.0–13.0 MHz) was used. All images were recorded using the extended field of view (EFOV) feature in the 10 MHz B mode. The EFOV image was used to measure the length of the Achilles tendon from the calcaneal notch to the musculotendinous junction of the gastrocnemius and this method has previously been found to be reliable and valid [28]. The participants were asked to lie down in a prone position with their hips and knees straight and their ankle hanging off the end of the bed. All examinations and measurements were performed by an experienced physiotherapist.

### Statistical analysis

All data were analysed using IBM SPSS Statistics for Windows (Version 23, IBM Corp., Armonk, NY, USA). Descriptive data are reported as the mean, standard deviation (SD) and range (min/max). The level of significance was set at *p* < 0.05. The limb symmetry index (LSI) was defined as the ratio between the involved limb score and the uninvolved limb score, expressed as a percentage (involved/uninvolved × 100 = LSI). The LSI was calculated and compared with 2-year results from the primary Achilles tendon rupture group [18]. A non-parametric test was used, as the data were not normally distributed. The healthy side was compared with the injured side using Wilcoxon’s signed-rank test. Effect size was calculated using Cohen’s *d*. To compare the re-ruptures with primary ruptures, the Mantel–Haenszel Chi-square exact test was used for ordered categorical variables, while the Mann–Whitney *U* test was used for continuous variables.

## Results

### Patient-reported outcome

**Fig. 2:**
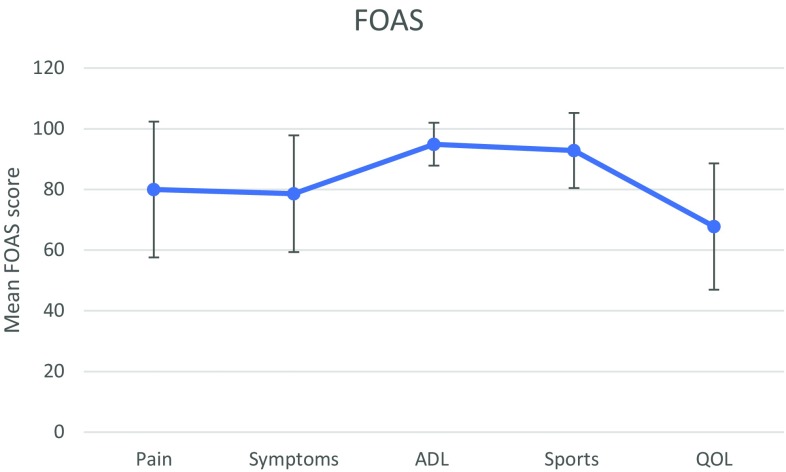
Foot and ankle outcome score profile

The mean (SD) ATRS was 78.4 (21) (range 32–100; median ± IQR = 89.5 ± 21). The median PAS score was 4 (range 2–6; mean (SD) 3.9 ± 1.7). On average, the patients reported having recovered 83.3% (11.7%) of full function (Table 2). Moreover, the five subscales of FAOS are presented in Fig. 2. The mean quality-of-life score was 67.8 (20.8).

**Table 2:** Table patient-reported outcome

Patient-reported outcome	Re-rupture (*n* = 20)
ATRS	78.4 (21.0)
	(68.5; 88.2)
	*n* = 20
PAS	3.9 (1.2)
	(3.4; 4.5)
	*n* = 20
Percentage recovery	83.3 (11.7)
	(77.8; 88.7)
	*n* = 20

For continuous variables mean (SD)/(95% CI for mean)/n = is presented

*ATRS* Achilles tendon total rupture score, *PAS* physical activity scale

### Functional tests

The injured side had significant deficits compared with the healthy side in terms of heel-rise functional measurements, except for heel-rise work (Table 3). However, the effect sizes indicate that these differences were relatively minor.

**Table 3:** Performance on functional tests for the injured versus uninjured side

Functional outcome	Injured side (n=20)	Healthy side (n=20)	Comparison between injured side and healthy side		
			Mean difference	Effect size	*p* value
Hopping height (cm)	4.13 (1.73)	4.11 (1.92)	− 0.02 (0.99)	0.01	n.s
	(3.32; 4.94)	(3.21; 5.01)	(− 0.48; 0.48)		
	*n* = 20	*n* = 20	*n* = 20		
Hopping polymetric quotient	0.53 (0.14)	0.54 (0.18)	0.01 (0.1)	0.03	n.s
	(0.47; 0.6)	(0.46; 0.62)	(− 0.04; 0.05)		
	*n* = 20	*n* = 20	*n* = 20		
CMJ height (cm)	12.6 (5.3)	13.5 (5.1)	0.88 (2.67)	0.17	n.s
	(10.1; 15.1)	(11.1; 15.8)	(− 0.37; 2.12)		
	*n* = 20	*n* = 20	*n* = 20		
Drop CMJ (cm)	13.2 (5.5)	15.1 (6.3)	1.90 (4.12)	0.32	0.039
	(10.7; 15.8)	(12.2; 18.1)	(− 0.03; 3.83)		
	*n* = 20	*n* = 20	*n* = 20		
Concentric power (W)	301.9 (181.8)	319.3 (227.6)	17.4 (147.6)	0.08	n.s
	(216.8; 387.0)	(212.8; 425.8)	(− 51.7; 86.5)		
	*n* = 20	*n* = 20	*n* = 20		
Eccentric-concentric power (W)	368.0 (231.4)	322.5 (133.2)	− 33.8 (182.9)	0.24	n.s
	(256.4; 479.5)	(260.2; 384.8)	(− 121.9; 54.4)		
	*n* = 19	*n* = 20	*n* = 19		
Heel-rise repetitions (n)	29 (14)	32 (13)	3 (5)	0.24	0.004
	(22; 35)	(26; 38)	(1; 5)		
	*n* = 19	*n* = 19	*n* = 19		
Heel-rise work (J)	1960 (830)	2320 (770)	360 (660)	0.45	n.s
	(1560; 2370)	(1950; 2690)	(40; 680)		
	*n* = 19	*n* = 19	*n* = 19		
Heel-rise max height (cm)	11.9 (1.9)	12.5 (1.5)	0.68 (1.22)	0.35	0.0078
	(11.0; 12.7)	(11.8; 13.3)	(0.11; 1.24)		
	*n* = 20	*n* = 20	*n* = 20		

For continuous variables mean (SD)/(95% CI for mean)/*n* = is presented

*CMJ* counter movement jump

### Clinical outcome measurements

When comparing the injured and healthy sides, the injured ankle had a significantly smaller degree of dorsiflexion with the leg extended (*p* = 0.003), but there was no difference when the knee was flexed (Table 4). Calf circumference was significantly greater on the healthy side (*p* ≤ 0.001) compared with the injured side (Table 4). No significant differences could, however, be identified in terms of tendon length or ATRA angle between the two sides (Table 4).

**Table 4:** Clinical measurements for the injured versus healthy side

Clinical measurements	Injured side (*n* = 20)	Healthy side (*n* = 20)	Comparison between injured side and healthy side		
			Mean difference	Effect size	*p* value
ATRA (°)	55.9 (5.8)	55.9 (5.2)	0.000 (4.280)	< 0.01	n.s
	(53.2; 58.5)	(53.4; 58.3)	(− 2.003; 2.003)		
	*n* = 20	*n* = 20	*n* = 20		
Tendon length (cm)	22.5 (2.5)	21.8 (2.8)	− 0.679 (2.124)	0.26	n.s
	(21.3; 23.7)	(20.5; 23.2)	(− 1.703; 0.344)		
	*n* = 19	*n* = 19	*n* = 19		
Dorsiflexion ankle with extended leg (°)	35.3 (8.7)	40.8 (7.4)	5.51 (7.46)	0.68	0.003
	(31.3; 39.4)	(37.4; 44.3)	(2.01; 9.00)		
	*n* = 20	*n* = 20	*n* = 20		
Dorsiflexion ankle with flexed leg (°)	38.1 (7.9)	44.3 (9.6)	6.23 (13.19)	0.7	n.s
	(34.4; 41.8)	(39.9; 48.8)	(0.05; 12.40)		
	*n* = 20	*n* = 20	*n* = 20		
Calf circumference (cm)	37.1 (2.2)	38.4 (2.2)	1.33 (1.20)	0.59	< 0.001
	(36.0; 38.1)	(37.4; 39.4)	(0.77; 1.88)		
	*n* = 20	*n* = 20	*n* = 20		

For continuous variables mean (SD)/(95% CI for mean)/*n* = is presented

*ATRA* Achilles tendon resting angle

**Table 5:** Patient-reported and functional outcome of re-ruptures versus primary ruptures

	Re-rupture (*n* = 20)	Primary rupture (*n* = 81)	*p* value
Patient-reported outcome			
ATRS	78.0 (21.2)	89.5 (14.6)	0.007
	(68.1; 87.9)	(86.3; 92.7)	
	*n* = 20	*n* = 81	
PAS	3.90 (1.17)	3.76 (0.95)	n.s
	(3.35; 4.45)	(3.55; 3.96)	
	*n* = 20	*n* = 81	
Functional outcome			
LSI heel rise work (%)	86.2 (29.1)	81.2 (18.6)	n.s
	(72.2; 100.2)	(77.1; 85.3)	
	*n* = 19	*n* = 80	
LSI heel rise rep (%)	88.0 (18.6)	97.7 (16.7)	n.s
	(79.0; 97.0)	(94.0; 101.5)	
	*n* = 19	*n* = 80	
LSI heel rise height (%)	94.7 (9.3)	83.5 (11.7)	< 0.0001
	(90.4; 99.1)	(80.9; 86.1)	
	*n* = 20	*n* = 80	
LSI CMJ (%)	94.7 (17.6)	91.9 (14.8)	n.s
	(86.4; 102.9)	(88.6; 95.2)	
	*n* = 20	*n* = 81	
LSI concentric power (%)	93.5 (38.9)	86.1 (32.9)	n.s
	(75.3; 111.7)	(78.7; 93.4)	
	*n* = 20	*n* = 79	
LSI eccentric-concentric power (%)	110.4 (49.8)	79.3 (21.0)	0.001
	(86.4; 134.4)	(74.5; 84.0)	
	*n* = 19	*n* = 78	
LSI drop CMJ (%)	89.2 (22.3)	88.7 (16.3)	n.s
	(78.8; 99.6)	(85.1; 92.3)	
	*n* = 20	*n* = 80	

For continuous variables mean (SD)/(95% CI for mean)/*n* = is presented

*CMJ* counter movement jump, *DJ* drop jump, *ATRS* Achilles tendon resting angle, *PAS* physical activity scale

### Comparison with 2-year data from primary ruptures

The groups were comparable in terms of demographic data. Re-ruptures had a significantly worse mean (SD) ATRS score 78 (21.2) compared with 89.5 (14.6) (*p* = 0.007), (Table 5). However, heel-rise height (*p* ≤ 0.0001) and eccentric-concentric power (*p* = 0.001) were better in the re-rupture group compared with the primary rupture group. No other functional tests revealed significant differences between the groups (Table 5).

## Discussion

The most important finding in this long-term follow-up was that patients with an Achilles tendon re-rupture were significantly affected by the injury. Patient-reported outcome scores were low in terms of both the ATRS and FAOS and only two patients reported a 100% recovery. Functional deficits were present on the injured side compared with the healthy side. In comparison with primary ruptures, patients with a re-rupture had lower patient-reported outcomes; however, in contrast, they had similar or superior functional results.

The subjective functional complaints can also be demonstrated by the functional outcome measurements. In comparison, the heel-rise height was worse compared with the healthy side. Previous studies have shown reduced plantar flexion strength after a re-rupture [21, 22], in contrast Metz et al. [11] did not find deficits in strength. It has been postulated that this difference was due to the difference in follow-up period, with Metz et al. having a mean follow-up of 9 years. However, a 7-year follow-up of primary ruptures recently published by Brorsson et al. [2] shows that no significant recovery occurs after the first 2 years. In the present study, there were no significant differences in either concentric, eccentric-concentric power or heel-rise work compared with the healthy side, demonstrating minor functional deficits in terms of strength. Moreover, significant functional deficits were found in terms of heel-rise repetitions, heel-rise height and drop CMJ compared with the healthy side. The largest difference between injured and healthy side was found in heel-rise work, however, the effect size indicates the difference is still minor. It is clear that re-ruptures have a inferior functional outcome in the injured tendon compared with the healthy tendon and that there is a large variation between patients. This indicates that some patients recovered well, whereas others made an unsatisfactory recovery. A similar picture is found in primary ruptures. The reasons for this need to be further explored.

Various surgical techniques for treating re-ruptures have been described, with advantages and disadvantages reported for each procedure [15]. The limited number of patients included in all the previous studies, together with heterogeneous, non-validated outcome measurements, make it difficult to compare the different surgical methods. Using a free gastrocnemius aponeurosis flap, as in the present study, has the advantages of being easy to perform and only one incision was used. One major limitation with this technique is that it can only be used for gaps of less than 6 cm [15]. This technique appears to be beneficial when it comes to restoring heel-rise height and tendon length. However, the operated side had reduced range of motion, which is not the case when it comes to primary ruptures. It can be hypothesised that the stiffness causes the continued symptoms and this may correlate to the psychological trauma caused by sustaining a re-rupture. Deep adhesions following extensive surgery may also lead to increased stiffness [12, 17]. A recent study by Silbernagel et al. [29] showed that tendon elongation is correlated with deficits in heel-rise height between the injured and healthy sides. In the present study, the mean length of the injured tendons compared with the healthy side revealed that some elongation appears to be present; however, it did not reach a significant level and might explain the minor difference in maximal heel-rise height.

The re-ruptures were compared with 2-year follow-up data from a previous RCT [18] comparing surgical and non-surgical treatment. As stated above, it was recently shown that no significant improvement occurs after 2 years [2] and we therefore, concluded that this comparison would reflect the long-term outcome for primary ruptures. Interestingly, the re-rupture group had a worse patient-reported outcome, but the functional outcome was very similar or even superior in terms of heel-rise height and eccentric power. As heel-rise height is a reflection of the length of the tendon [7], this possibly shows that the surgical technique used for the re-rupture group is superior when it comes to maintaining the length of the tendon compared with the treatment protocols for the RCT. The FAOS score showed that re-ruptures obtain poor quality-of-life scores compared with primary ruptures, indicating that these patients are severely affected by their injury. The poorer patient-reported outcome is probably due to the severity of the injury and the prolonged psychological impact, as it is not reflected in the functional outcome. Patients with re-ruptures may need closer contact with their physiotherapist to improve their outcome and their quality of life during the follow-up period.

The present study population with 20% women with a mean age of 41.5 (16.2) is a representative sample compared with previous RCTs in terms of demographics [14, 20]. Recent studies of predictors of outcome have shown that being female, older age and a high BMI are associated with an inferior outcome [19, 26]. However, this interesting topic is still open to debate, as Arverud et al. [1] presented conflicting findings, with being male predicting a poorer outcome. We were unfortunately not able to conduct a gender-based sub-analysis, as the present study includes too few patients. The gender difference in terms of both primary ruptures and re-ruptures needs to be explored further in larger patient cohorts.

The findings in this study raise questions that require further research. It is of great interest to understand why there is a discrepancy between the patient-reported outcome and the results of functional testing within the re-rupture group, which are not present in the primary rupture. The mechanism behind this needs to be studied and explained. The current treatment for re-ruptures yields acceptable results, but there are still significant deficits in functional outcome, which need to be addressed. The obvious limitation to this study is the small number of patients. This makes it difficult to draw any strong conclusions. Re-ruptures are fortunately uncommon, as previously reported, which makes it difficult to include a large number of patients. However, to our knowledge, this is the largest cohort of reported re-ruptures. The strengths of the present study are the use of a strict protocol using validated, well-documented outcome measurements and the fact that all the data were collected by the same experienced physiotherapist. The comparison group was also evaluated using identical methods.

## Conclusion

The results of this study indicate that patients with an Achilles tendon re-rupture have continued symptoms and functional deficits on the injured side. Patients with an Achilles tendon re-rupture had worse patient-reported outcomes but similar or superior functional results compared with patients with primary ruptures.
